# Towards Cognitive Authentication for Smart Healthcare Applications

**DOI:** 10.3390/s22062101

**Published:** 2022-03-09

**Authors:** Ali Hassan Sodhro, Charlotte Sennersten, Awais Ahmad

**Affiliations:** 1 Department of Computer Science, Kristianstad University, 291 88 Kristianstad, Sweden; charlotte.sennersten@hkr.se; 2 Shenzhen Institutes of Advanced Technology, Chinese Academy of Sciences, Shenzhen 518000, China; 3 Department of Computer and System Science, Mid Sweden University, 831 25 Ostersund, Sweden; awais.ahmad@it.uu.se

**Keywords:** cognitive authentication, IoT, healthcare, EEG, biometrics, sensing

## Abstract

Secure and reliable sensing plays the key role for cognitive tracking i.e., activity identification and cognitive monitoring of every individual. Over the last years there has been an increasing interest from both academia and industry in cognitive authentication also known as biometric recognition. These are an effect of individuals’ biological and physiological traits. Among various traditional biometric and physiological features, we include cognitive/brainwaves via electroencephalogram (EEG) which function as a unique performance indicator due to its reliable, flexible, and unique trait resulting in why it is hard for an un-authorized entity(ies) to breach the boundaries by stealing or mimicking them. Conventional security and privacy techniques in the medical domain are not the potential candidates to simultaneously provide both security and energy efficiency. Therefore, state-of-the art biometrics methods (i.e., machine learning, deep learning, etc.) their applications with novel solutions are investigated and recommended. The experimental setup considers EEG data analysis and interpretation of BCI. The key purpose of this setup is to reduce the number of electrodes and hence the computational power of the Random Forest (RF) classifier while testing EEG data. The performance of the random forest classifier was based on EEG datasets for 20 subjects. We found that the total number of occurred events revealed 96.1% precision in terms of chosen events.

## 1. Introduction

The key goal of the cognitive authentication is to identify, trace and track individual differences of biological signatures, behavioral features such as facial expressions, fingerprints, voice, eye movements, gestures, and postures, etc. [1,2]. At present numerous emerging strategies related to the biometric authentication are being integrated and evolved in interdisciplinary domains i.e., personal identification, access and assets monitoring applications. Among biometric-based technologies it is witnessed that EEG signals gathered during behavioral and/or mental activity can be adopted for reliable authentication and identification [3]. On one hand EEG enabled systems are contributing into the human identity monitoring field, while at the same time we are facing various critical challenges such as security, accuracy, privacy, and robustness, etc. To fix these concerns it is very vital to develop innovative and secure methods to protect and secure the individual privacy [4]. The Human Computer Interface (HCI) is playing a catalyst role while displaying stimuli (visual or auditory) and capturing signals recognizing the personal identity trait. There are still large research gaps regarding security, privacy and energy efficiency. Resolving emerging problems and trends in association with strong ties of Brain Computer Interface (BCI) such as virtual headsets and internet are key parameters in personal authentication and recognition [5,6].

Traditionally security methods are not up to scratch and effective when looking at simple legalization and identity monitoring issues. So, to cope with these problems an EEG based physiological system is a suitable candidate for the upcoming next generation systems [7,8] due brainwaves reflect/mimic the human mode and actions performed. The secure, prominent and malware detection capabilities of brainwaves make EEG i.e., brainwaves unique and attentive for many fields especially wireless networks, various industries, and academia. Few decades ago, some researchers worked and addressed the emerging role and importance of brain signals while identifying and monitoring the activity of the individual entities [9]. Biological features are key since long time and emphasized by researchers and academics [10]. Brainwaves are related to the neurons of the human showing various brain activity in regular and random ways [11]. Due brainwaves’ strong connection and association with mental tasks, their states are hard to interpret, copy and hijack from external source if used as passwords. Moreover, EEG signals are interlinked with someone’s thoughts, knowledge, and memory which makes it hard for someone to exploit, steal and replicate [12,13]. EEG reflects and contains the information from human neurons via small, more effective, and lightweight devices [14,15,16]. To fulfill and meet the demands of public citizens it is to provide a highly secure and energy efficient protective, flexible, scalable, unique, and stable biometric system to compensate for the drawbacks of current conventional platforms [17,18]. However, EEG performance suffers in some fields due to noise, interference, and compatibility issues. EEG enabled systems still need higher maturity and improvement in high-level security, comfort, and ease to end users. Therefore, it is very important to propose a novel, and unique EEG enabled biometric platform for real time or near real time healthcare applications. This paper reviews, addresses current trends and practices in the field of EEG based biometric systems and discusses the key merits, demerits with a recommended tentative future solution.

Authentication is considered as the key part in computer platform security, and this prevents access by hackers and illegal users. In addition, the process is to match or check the similarity and coherence between stored and obtained data. In an agreement condition, a person or a computer is potentially allowed to access the system else prohibited. It is important to have the accurate authentication rights to access the system. The result of a non-secure system is information privacy leakage and breaches which will further result harming the customer. Today’s most powerful authentication systems have password protection and identity monitoring. Users make themselves recognizable via words/passwords, but this let alone is not enough to fully identify and protect personal information. The traditional password protection is lacking in cognitive uniqueness therefore anyone can steal and hack it. To advance a poor password and keep it secure the user needs to increase the complexity-level which will be very hard to memorize. To progress and replace current orthodox security techniques, it is suitable to introduce and address the notion of biometric authentication. Tangible features as fingerprints, voice and facial expressions are hard to be compromised and stolen by attackers and intruders. Thanks to tangible brain readings recorded via EEG, it creates strong unique identity traits hard to replicate. It is observed and analyzed that other physiological features such as Electrocardiography (ECG) is easy to attack and exploit.

The main contribution of this paper is to present an extensive review about a secure, reliable cognitive/EEG-based authentication system for the IoT-driven healthcare applications as revealed in Table 1. The paper analyses 20 subjects’ EEG datasets imported from the Physionet bank ATM database and validates the performance of the proposed framework in terms of performance indicators via accuracy, precision, true positive (TP) rate and precision with the help of the Random Forest (RF) classifier as shown in Table 2. In addition, this research focuses on EEG-based biometric systems, their advantages, limitations, applications, and to propose a novel framework approach.

The paper is organized as follows. Section 2 presents an extensive review of current work, further in Section 3 a secure and reliable cognitive/EEG-based authentication system is presented. Section 4 proposes the novel framework for the same system. Methods of the system are addressed in Section 5. Detailed applications of are explored in Section 6. After in Section 7 tentative solutions and recommendations are presented. EEG data collection and experimental setup is presented in Section 8. Last in Section 9 a conclusion of the paper.

## 2. Existing Work

In the following section, several papers by various researchers are listed and compared in relation to focus and respective traits with the aim to provide a secure solution for public. With respect for all individual efforts there is still room for innovative solutions using EEG. Some of the relevant research contributions are listed, what focus and crosschecked in Table 1 below.

First part of this extensive survey presented the security and privacy related techniques, solutions, and challenges, while the second part was mainly concerned with the biometric enabled systems and their comparisons to the traditional platforms, limitations, and advantages. Thirdly, proposes the tentative solutions, and recommendations.

## 3. Deficiencies in Authentication Methods in IoT-Based Healthcare Applications

Current traditional human physiological features are not satisfying the needs for several domains i.e., medical hospitals, industrial platforms, and academia. In comparison to the traditional password authentication process, the biometric tracking is more feasible and unique with its physiological features. The output from the human body parts result in reduced efforts memorizing extra passwords and security codes. The biometric input tracking from human is given below:

- Fingerprint scans: Scans and maps fingers.
- Handprint scan: Tracks and scans the entire pattern of the hand.
- Voice identification: Recording human voice signals and traits.

Some important steps must be taken into consideration though while introducing the concept of real-time applications.

For a system of this kind, it needs some common prerequisites to perform to meet individual unique needs;

- Universal: Monitoring the identity of the system-users we need biological traits to minimize the chances of information leakage.
- Uniqueness: To differentiate every human physiological feature from others to achieve an accurate error free result.
- Stability: There should be less variation and more accurate outcome.
- Flexibility: Adaptive and scalable in nature regarding the physiological features. As a result, it will be hard decoding and leaking the secret information.
- Acceptability: Important is to get individual consent for tracking and storage due private nature.
- Durability: Biological features are harder to replicate and this way also more sustainable dealing with security issues.

As we have already said EEG based authentication is widely adopted, precise, secure, and broadly accepted in the Security domain. The EEG activity mirrors different modes such as anger, happiness, crying, sadness, etc. Every single trait is unique therefore has a separate impact on entire individual signature performance. Mental activity and performed actions are replicated and mirrored in the EEG and works as signifiers during data analysis. These mental tasks have close and strong ties with the produced EEG patterns which serve to be more secure, and this way keep away a common intruder or un-authorized entity. Hence, in this situation it can be said that EEG based biometric is the two-step authentication and integration process. There are several other fields where EEG has remarkably played the major role in providing the security and innovative practices, that is why EEG based features outperforms the others. Most importantly, EEG or brainwaves are related to the human neurons so when these are active and functioning properly than several signals and information types can be exchanged. In other words, we can say that EEG is a more prominent communication pattern identifier unlike other biological signals.

In case of emergency, injuries, and other accident issues the biometric pattern(s) can be affected and people are in this case not allowed to attend personal authentication until their injuries are healed. One hazard and limitation, fingerprints can still be performed in a state of dizziness and inactive mode. If fingerprints are cross checked with the EEG signal though it is still hard to be hijacked and leaked. EEG based techniques in comparison to fingerprint authentication is facing a critical challenge of Gaussian noise which can affect sensitive healthcare information. This means we must be aware of both pros and cons from embodied electrodes/leads.

## 4. Proposed Framework of Cognitive Authentication System

**Figure 1:**
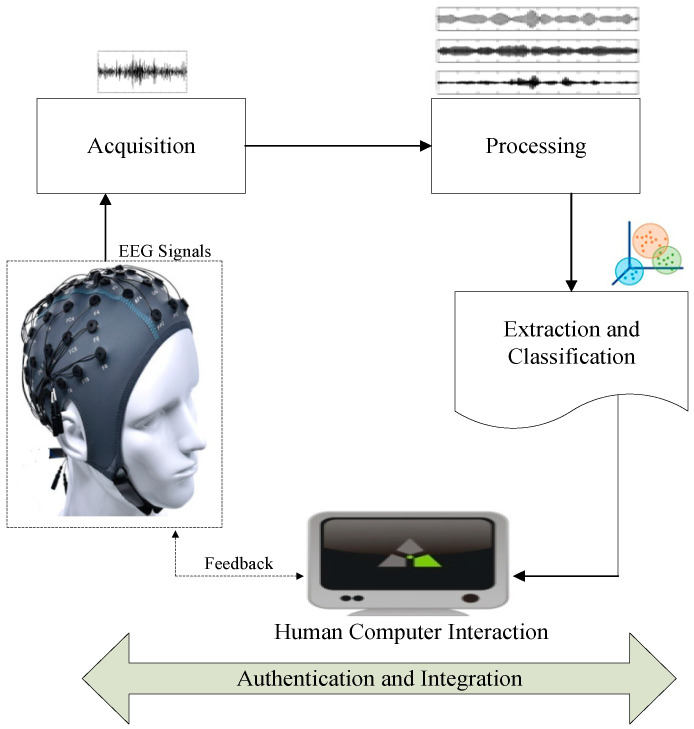
Proposed Cognitive Authentication Framework for smart Healthcare Applications.

We would like to propose a novel cognitive authentication framework comparing various core concepts of privacy and security provisioning shown in Figure 1. This cognitive authentication framework of cognitive/EEG signals are processed, extracted and classified by adopting machine and deep learning based on adaptive methods. After human computer interaction (HCI) is established the feedback from HCI via the original EEG signal plays a significant role when managing and monitoring the brain wave authentication. Our proposed framework is most simple, efficient, and secure for the medical healthcare sector applications facilitating the patients and physicians at an economical rate. We aim to develop a prototype of brain wave monitoring under strict security assurance. The key characteristic of the proposed framework follows. To limit complexity a dynamic self-identity management mechanism i.e., biometric system can be developed merely with the EEG signals. From previous research results we realize there is no mature nor practical biometric system of self-adaptive nature character i.e., security and energy optimal. In this regard, it is vital to have an EEG-based cognitive ideal authentication system allocating parameters in an efficient and authenticated way.

### 4.1. Simple and Efficient

It is observed and analyzed from previous research studies that high performance can be achieved from a well deployed and secure system [15,16,17,18,19], while with a more computational complex task(s) system a threshold is met due to more resources e.g., time consumption and longer delay. The authentication, balances between individual entities on one hand which on the other hand affects other parts of the system.

### 4.2. Channels and Electrodes

Numerous types of EEG headsets are adopted for exact and accurate measurements to provide high level security in both clinical and academic contexts. It is proved that as the number of leads/channels are increasing than higher and better accuracy will be achieved [11]. With high accuracy there are less chances of information hack and eavesdropping. A strong presence of security level is a prioritization. A simple and less probes-based EEG system takes less computational power and results in compromised security, that is why not a potential candidate [15,16,17,18,19,20]. Researchers [21,22,23,24,25], propose wet EEG electrodes for high signal accuracy, calibration and less noise, interference unlike the dry leads.

### 4.3. Data Collection Pattern

Large data sets enhance the accuracy of the adopted algorithms. Researchers in [12] examine the voluminous datasets into single and multi-patterns. Authors in [14] examine categorization of different subjects based on different factors for example, maximum number of participants, accuracy of the system and outcome. Systems with data from few subjects are not robust. Researchers in [20] present data gathering tools based on several subjects with their unique features and problem-solving traits.

### 4.4. Computational Cost

Computational cost is related to several techniques and parameters such as number of electrodes, thinking responses, heartbeat fluctuations, behavior analysis, and neural and fuzzy based machine learning methods, etc. Integration of classified traits extend and promote accuracy levels even if longer delays.

### 4.5. Stability

Cognitive/EEG or human brain waves are dynamic and well pattern-based mechanism with adaptive nature. In addition, the action and activities are revealed according to priorities and choices of humans towards training [23] of their mind. Most of the time real-time data sets are helping to identify the behaviors and ties in terms of his/her consistency and attitude [25]. In the long run data sets are not suitable candidates analyzing an entire behavior of a human. Live human actions in combination with mental tasks are more promising and relevant fully incorporating the desired tasks.

### 4.6. Flexibility

Traditional biometric based systems are hard to change due to their close association with the human biological features unlike the password-protection based on secure and authenticated attributes. In the present era, EEG enabled biometrics has created huge attention worldwide according to the advancements of capturing human brainwaves and mental task readings [11].

## 5. Methods for Cognitive Authentication System

There are several methods for security and privacy preserving data and information but biometrics is the best suited method to deal with the vulnerability problems. Here we present some of the techniques providing efficient methods.

### 5.1. Machine Learning

Self-adaptive and intelligent techniques are the key ingredients to revolutionize the digital world without the intervention of the external features and resources. The machine learning (ML) driven techniques are highly dependent on the datasets by analyzing and examining the critical challenges for instance, linear regression, hierarchical, and clustering etc for the clear understanding of desired target. So, this sub-section highlights the importance of intelligent techniques in portraying the clear characteristics of EEG to evaluate the disease type. After careful examination and importance of the self-driven mechanism ML is separated into following keys methods.

⮚ Supervised learning: In this method tags are assigned to specific data types by classifying main groups/category(ies). Besides, data patterns are extracted based on preliminary data models by properly guiding the future techniques.
⮚ Unsupervised Learning: There is no particular label to the clustered data sets due to the self-learning learning nature of the data-driven model. In addition, data patterns are recognized based on previous gained knowledge.
⮚ Reinforced Learning: The key characteristics of this data model is to communicate with external entities by collecting and enhancing the knowledge. Then a reward or penalty will be assigned based on the action (i.e., success/failure) taken accordingly. Furthermore, data models are trained and analyzed by adopting two intelligent sub-areas such as, support vector machine (SVM) and deep learning (DL). Both SVM and DL follows the supervised learning strategy to further improve the performance of entire system. In last, this learning mechanism helps the brain to analyze the EEG behavior in a better, self-adaptive and effective way.

For further understanding the clustering and classification challenges in data models such as SVM is considered as the key role player. Data sets are spread and adopted in multi-dimensional areas with number of rows and columns. While this paper considers the EEG as an evolutionary mechanism for understanding the human brain activity. SVM is involved to build hyper-planes and by classifying and categorizing the data/data models, the hyper-plane is very vital to compose the desired datasets. In the bunch of data sets one hyper-plane is enough to lead the entire group. Wise selections of hyper-planes give better options to play with datasets managing, monitoring, and classifying them into intendable and interpretable platform outcomes. Furthermore, hyper-planes are the key ingredient to deal with the datasets in this intelligent data world.

### 5.2. Deep Learning and Neural Network

Deep learning (DL) lies under the umbrella of machine learning, and it is used to model accurate and efficient modelling of random and abstract data content(s). The DL adopts less computational steps to complete a task with short time span unlike traditional ML methods, so it is preferred in most research. DL foundations are also key steps toward an artificial human nervous system integrated with robotics and self-driven fields. Besides, a multi-layer hierarchy is adopted in the entire system with its input from previous layer as output of the next coming one, this will continue up to the intended outcome with minimum error. All the interconnected layers exchange the information among each other and other associated entities at lower and higher levels through the specific functional units, neurons. Most of the interconnected entities i.e., neurons function by adapting vital procedural parameters from well-known DL models for instance, deep neural networks (DNN), convolutional neural networks (CNN), and recursive neural networks (RNN). In this research DNN is considered as the game changer and potential candidate to tackle the entire process in EEG monitoring, clustering, and management.

Our targeted DNN platform comprises input, output, and hidden layers to form the long lasting and collaborative hierarchical network for data exchange with specific processing units and task allotment. Each layer is characterized with the number of nodes to fulfill the criteria of the network by properly designing the entire platform with slightly more deviation in the hidden layer format with distinct features. In addition, a hidden layer plays the major role in analysing the computationally complex process of several inputs and outputs.

### 5.3. Self-Adaptive and Dynamic Resource Allocation

Using the methodology of neural network and fuzzy logic the resources such as, battery lifetime and power can be allotted to medical devices in a fair and intelligent way. Security is the major challenge for EEG devices, power and battery lifetime results in less storage space for these devices.

In this regard compressive sensing is the potential candidate in most of the traditional platforms, but still there are several limitations while dealing with EEG based biometric environment. So, to remedy most of the issues chaotic compressive sensing is a suitable option.

## 6. Applications of Cognitive Authentication System

There are various applications of EEG-based cognitive authentication systems, some widely scoped and discussed one by one below.

### 6.1. Medical Healthcare

Pervasive nature of mobile devices in healthcare domain face hard challenges while securing the sensitive information between various entities from patients to physicians and from medical clouds to hospitals. In other words, we can say that there is a need of novel EEG based biometric techniques to fix both the energy efficiency and security issues.

### 6.2. Industrial Enterprises

In today’s emerging era of the forth industrial revolution, Industry 4.0, there is a need of highly demanded security and authentication methods to deal with the security and management issues of voluminous data from different industries. If customers are not getting proper security while buying any product it will be hard to gain their trust. Thus, product owners must guarantee valuable security and efficient methods.

### 6.3. Access Control and Personal Identity Management

Personal security and identity monitoring is the first and foremost priority of everyone signing any contract, agreement, or bond with anyone, anywhere. Without proper trust and belief one cannot assign, handover his/her property at risk. Specially, in the healthcare domain it is very vital to respect and protect patient’s secret and sensitive data and keep it away from any harm. Hence, it is important to propose novel access control for personal data/information management.

## 7. Advantages, Limitations, Tentative Solutions and Recommendations

This section presents the novel tentative solutions with potential recommendations for solving the security issues in most of the areas especially medical information communication technology (MICT) for healthcare.

EEG-based cognitive systems with biological features are key players in selecting a suitable protocol to monitor and identify the identity of individuals for the better and strong security levels. Noise and interference occurs between neurons while adopted for human identity recognition due to separated and unique roles for each biological trait in the biometric system. Moreover, collected random neurons are associated with the mental tasks and superimposed behavior of other similar functional brainwave signals in speeding up the entire process. Physiological features such as eye movements, hear-rate, blood circulation are a little bit different from the EEG signals. The huge and bulky load of brain responses, the interference and noise levels are relatively low while recording the EEG signals. Thus, some careful and efficient steps must be taken to remedy most of the unwanted noise in the very sensitive information throughout the system. The main beauty of the EEG based signals for biometrics is that there is slightly less noise and artifacts [42]. The downside of EEG enabled biometrics is that the security level is less reliable when low-cost hardware devices are adopted. These security challenges are part of practical issues in the healthcare domain [11].

EEG based systems are not new, also widely portrayed in various domains since long time. Some key problems faced while deploying such system, there are no direct off the shelf suitable tools and software, second, more computational complexity, and delay in transmitting/exchanging information to intended users.

### 7.1. Advantages of Cognitive Authentication System

Emerging trends and rapid proliferation in the technological EEG-driven cognitive authentication systems era emit every living being unique brain pulses for stimulating the visual and auditory sections. EEG maps the signal of brain activity related to either (1) screen stimuli or (2) thought process. If gaze is used in addition to a thought process, then we can add these screen coordinates as an additional authenticator for security reasons. If a person looks at a stimulus it generates both an EEG signal via the electrodes and also a gaze pattern via gaze tracking. The latter shows what a person is looking at on the screen while the EEG signal indicates a COS/SIN curve which does not reveal anything of gazed location on the screen nor the gazed content. The abstract thought processes of thinking, emotions, dreams, perceptions, feelings, levels of happiness and sadness all impact on the outcome of mental process generating unique EEG patterns accordingly. In an event-triggered situation, the critical brain waves will show different responses in comparison to a normal situation. These various patterns are hard to crack if anonymous to the intruder. Moreover, EEG based security methods are more robust to handle hacking and eavesdropping as compared to conventional methods. In case of illegal and forceful authentication by intruders and attackers, the security level will not be compromised. Therefore, it can be said that other less secure and ineffective methods are easy to be interpreted and compromised and not reliable and sustainable entities in a security provisioning domain [8]. In other words, it can be said that EEG signals are built-in, or by-default featured for high level security and are hard to comprise as compared to other technologies [6]. Due to simple, effective ways, they are hard to decode. These technologies revolutionize the biometric platform era with high end security patterns in combination.

### 7.2. Limitations of Cognitive Authentication System

The EEG signal capturing devices are low power oriented which is not a limitation itself. Pre-processing is more sensitive to handle due fast paced feedback responses happening in a few microseconds. Most of the current technologies are leads/electrodes integrated for calculating the EEG signals with non-invasive nature. However, the responses recorded by an electrode varies to a large extent even if its position deviates by a minuscule pattern [29]. Electrodes must be implanted in a careful, exact, and proper manner while providing high authentication for a system. Consistency between EEG signals before and after examination is important to take under consideration [30].

Subjects with both physiological and mental conditions and their data readings must be synchronized to meet a sustainable and stable accurate baseline performance maintaining high security level. The captured brainwaves reveal the mental task via continuous measurements. This means any slight change in mood or mental state will be reflected during measurements. To prevent and to create a calibrated baseline, it is required to keep a steady mental state prior EEG recording.

Therefore, it is preferred to put individuals in rest mode by closing their eyes and turn-off all unwanted sources to provide high level efficiency for collecting the accurate and desirable EEG signals. This way we can get eliminate data noise and disturbances.

### 7.3. Recommended Solutions

#### 7.3.1. Solution1: Chaotic Compressive Sensing Enabled Authentication Scheme

A chaotic random captured mental process is hard to interpret. A more secure mechanism is sought for. The simple non-random data generation technique is more promising to build a matrix for a strong security platform [43]. In addition, this procedure forms a unique matrix by well-defined and clear steps. Most popular example of a chaotic process is the chebyshev chaotic filters [50,51,52,53] for further data generation operation.

**Figure 2:**
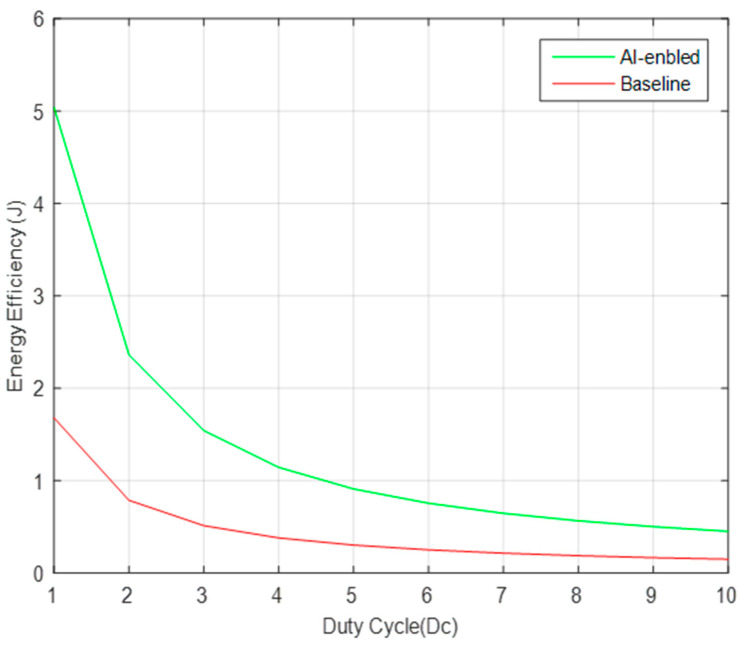
Energy Efficiency during compressive sensing in IoT devices.

The principle of this solution is based on the generation of a particular matrix encrypting the information, then transmitted over the wireless channel and finally decoded at the receiver side i.e., edge computing embodying higher security measures. The main advantages are to occupy less storage space, security and energy efficiency in the medical healthcare applications, as shown in Figure 2.

Efficient and voluminous storage space reduction are main traits of the compressive sensing providing a secure and energy efficient system in the healthcare environment [25]. Small size nodes communicate among themselves and neighboring nodes with help of intermediate routers i.e., relays up to shorter distance. Those relay nodes are necessary exchanging information between themselves (from-to), but it is not necessary to interpret and decrypt the messages from any outside device/relay. Only an authorized entity is allowed to decrypt/decode the message from a transmitting entity. Based on that message sharing capability using a sender-receiver pair, a measurement matrix will be generated to avoid information leakage and eavesdropping.

#### 7.3.2. Solution 2: Fuzzy-Vault Based Authentication Scheme

**Figure 3:**
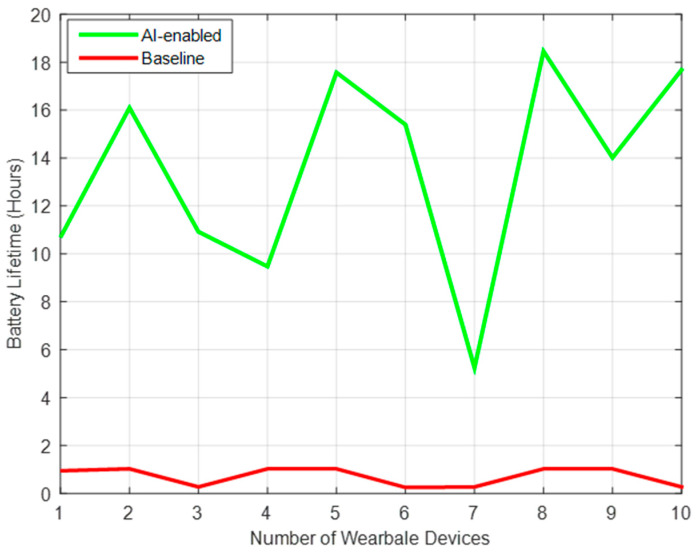
Battery Lifetime and Authentication level of IoT devices.

A random key is generated in a dynamic way unlike the traditional methods, so it is robust and accurate. This optimizes the resources in an efficient and autonomous way and will be widely used in the Bluetooth low energy domain including internet of medical things-based applications, as presented in Figure 3.

#### 7.3.3. Solution 3: Adaptive and Random Key Generation Mechanism

**Figure 4:**
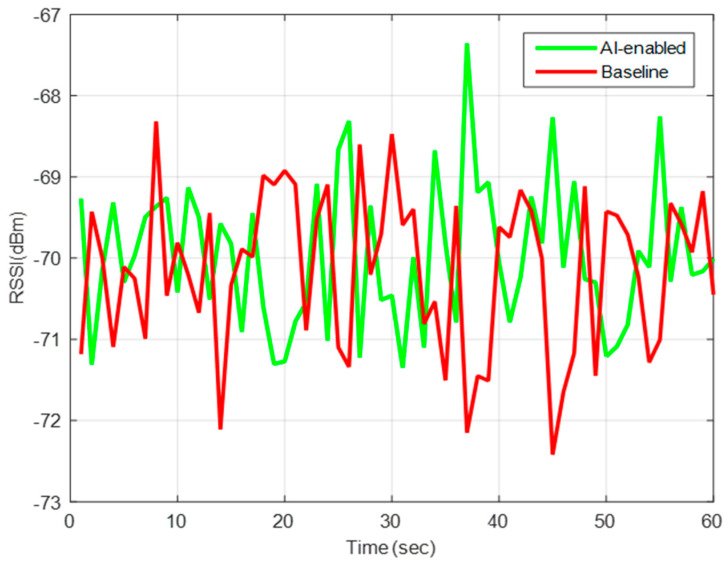
Reliability of IoT devices in terms of RSSI.

This solution can be effective and sufficient for the emerging mobile healthcare applications such as, Low Range (LoRa). The key mechanism of this approach is to generate the master key, which is eXclusive OR (XORed) with the certificate from a third trusted party. After receiving a certificate and a master key then an initial key will be produced protecting the entire system by adaptively adjusting the keys, as revealed in Figure 4.

## 8. Experimental Setup

To demonstrate the EEG based authentication method an experiment has been conducted to be able to showcase how we practically design, set up and use these measurements. Below are required steps including chosen datasets, events, classifiers, and results from these.

### 8.1. A. EEG Data Sets

We obtained EEG motor movement/imagery datasets from one of the largest and widely used databases called Physionet [112,113,114]. The selected datasets comprise 2 min indivudal EEG recordings of 109 subjects using the European Data Format (EDF) web browser. The data contains 64 scalp electrodes while performing a few tasks shown in the Table 2 below. In this experiment we considered only 20 subjects (s1, s2, to s20) out of total 109 data recordings.

One of the EEG subject scenario procedures are asking subjects to perform two actions at the same time i.e., reinforced activity consisting of opening and closing their right and left hand physically and at the same time mimicing the movements mentally. After this activity the subjects are asked to relax so baseline can be met.

### 8.2. B. Event Extraction

The recordings are in a comma-seperated-values (CSV) format containing Motor movement/imagery EEG data obtained from Physionetbank ATM and further analyzed via the European Data Format (EDF) browser. The main purpose to choose the EDF browser to obtain annotations and events during the experiments with temporailty, including start and total time of entire experiment. The chosen datasets carry three annotations which are time 0, time 1, and time 2 [T0, T1, T2] measuring the performance by included subjects. Notice all time related actions are performed as indiviudal isolated tasks. Further detail of the annotations are given as:

- T0 shows either (1) the event where the subject is in entire rest position or (2) performing any imagined motor movement task.
- The T1 event reveals the subject’s left-hand movement while performing either a physical or mental task.
- T2 is mainly reserved for observing subject’s right-hand movement either physically or mentally.

### 8.3. C. Classification

The EDF browser is used to achieve annotations for output labels so it is possible to run the classifiers; T0, T1, and T2 for each subject. For each classifier we have 64 electrode data points involved for effective and accurate classification as shown in Table 2.

The effective classification result is using the Random Forest method creating decision trees from training datasets in a random fashion [112,113,114]. The selection of an individual decision tree and different decision trees are the main decision factors of a final class of an object under test. The Random Forest classifier is adopted in our experimental setup because it is efficient and gives accurate estimation and classification with multi-inputs by large datasets.

### 8.4. D. Results and Discussion

The weighted mean of all included classes for each of the 20 subjects by considering the Random Forest Classifier for measuring the performance in terms of four main performance indicators for instance, accuracy, precision, true positive rate (TPR) and ROC area is presented in Table 2.

Precision: The small fraction of related events from the total number of occurred events in the experimental setup defines the precision. Suppose precision for S4 is 0.961, which reveals that 96.1% of chosen events are identical or related to each other as presented in Table 2.

True Positive (TP) Rate: The small number of positively corrected instances from total positive events gives the status of TP rate. One of the examples in Table 2 highlights the TP rate for S10 is 0.994 which means that events are classified with correction upto 99.4%.

Area Under Curve (AUC): For accuracy measurement of classifiers AUC is the appropriate indicator for test verfication, so higher the AUC means the better the test is. For instance, If AUC is 1, it means test of classifier is good and effective, while 0.5 shows poor or ineffective test. Table 2 shows the AUC value less than 0.7 for some subjects, and greater than 0.6 for most of the subjects. It is observed that test does not fail (less than 0.5) for any subject in our experiment which means Random Forest classifier is the potential candidate with better accuracy for 20 subjects.

Working through the cognitive authentication method and reviewing prior works, it is realized that any possible weaknesses such as possible falsification of data and/or online hijacking must be highlighted. Any data type, and number of features and signatures can be hard to replicate/mimic as previously said but even an inserted malware could possibly crack individual features over time. So, there must be potential solution for cognitive authentication with strong protective wall/firewall. In near future study we will run a cognitive authentication and try to hijack the authenticator/intruder with and without using firewall to check how long it takes to breach the security and reveal the identity. In other words, it can be safer if we connect cognitive authentication with two-factor authentication for strong security measure.

## 9. Conclusions and Future Research

This paper broadly presented the state-of-the art solutions and recommendations to fix the security and privacy problems by proposing the novel EEG-driven secure and reliable cognitive authentication system for a IoT-based healthcare system. An EEG-based cognitive authentication framework is proposed. Most remarkable and prominent EEG enabled biometric platforms are explored and addressed by both researchers and academics from practical aspects. It is analyzed and predicted from the broader scope and outcome of the EEG-driven biometric system that this can be adopted as a guide and trend setting solution for the next generation systems. It can be emphasized that future biometric environments must be developed in such a way that EEG channels/leads, algorithms, and signal processing techniques have strong ties with high level of coordination. By keeping in view, the demand of the healthcare in today’s world it is investigated that the energy optimization and security of nodes during information exchange are critical factors.

The key purpose of our experimental setup is to reduce the number of electrodes and hence the computational power of Random Forest classifier while testing EEG data. MATLAB is adopted for analysis and measuring the performance of the random forest classifier by testing EEG datasets of 20 subjects. We found that the RF classifier outperforms by revealing accurate and effective results, thus can be recommended for future similar scenarios and applications.

Hence, our future research will focus on the chaotic compressive sensing and self-adaptive i.e., machine learning strategies for the betterment of both security and power allotment in a fair way. In addition, the hardware, and the software platform for the chaotic compressive sensing for the e-Healthcare applications will be developed.

## Figures and Tables

**Table 1:** Related Works.

Ref. No.	Applications	Proposed Solution	Merits	Demerits
[1,2,3,4,5,6,7,8,9,10]	EEG, ECG, Secure BSN for medical care	Security and privacy	Energy efficient	Complex and inefficient
[10,11,12,13,14,15]	Smart, secure, and private media and bio-signal based Healthcare, IoT, physiological signals	Secure power control	Duty-cycle, data rate	High energy and battery drain during media transmission
[16,17,18,19,20]	Vital sign signals, security in cloud healthcare, EEG, ECG, medical IoT	Cloud and battery enabled	Fairy and battery efficient	Less power-aware and secure
[21,22,23,24,25]	WSNs, Secure and energy-aware IoT and BSN, EEG, vital sign signals	Frameworks and protocols	Extensive survey for healthcare	Not focused on mobile healthcare
[26,27,28,29,30]	Medical IoT, EEG, Data integrity and security in healthcare	Energy-aware and routing protocols	Energy optimization and efficient routing	Complex and less battery-efficient
[31,32,33,34,35]	EEG, Privacy in medical industrial applications, smart healthcare	Energy harvesting and duty-cycle enabled	Battery and energy-aware	Inappropriate for medical healthcare
[36,37,38]	EEG, medical IoT, Security and privacy in Telemedicine and BAN	QoS optimization based	Efficient QoS management	Less Battery and energy -efficient for healthcare
[39,40,41]	EEG, ECG, SpO2, smart and Secure healthcare, efficient Cellular networks	TPC and relay selection based	Novel Architecture and resource allocation method	High battery and energy drain in medical healthcare system
[42,43,44]	Private and secure communication systems	TPC and resource allocation	Energy optimization in wireless and sensor networks	Complex and less reliable for dynamic healthcare
[45,46,47,48]	ECG based secure BSN, Telemedicine, remote healthcare	Energy and battery-based frameworks and method	Efficient resource allocation	Complex and less battery-aware for medical services
[49,50,51]	Resource allocation in smart medical networks, EEG	TPC and radio-aware	Intelligent resource monitoring in radio networks	Unsuitable for healthcare system
[52,53,54,55,56]	Efficient and secure Future Networks, EEG, vital sign signals	QoS and Energy Scavenging	Novel energy and QoS efficient	Complex and less reliable for healthcare system
[57,58,59,60,61]	ECG, EEG, physiological signals, smart healthcare, IoT, lifecycle	TPC and QoS-aware framework	Detailed survey	Not focus at joint duty-cycle and TPC
[62,63,64,65,66]	Secure and cryptographic IoT for healthcare	Energy and battery-oriented	Novel Physical layer and framework for healthcare	Complex, less reliable without duty cycle
[67,68,69,70]	Green, battery-aware healthcare, BSN, medical IoT, ECG, EEG	Fuzzy based secure	Secure home monitoring	High energy drain
[71,72,73,74]	EEG, ECG, secure and pervasive WSN	TPC and battery-based	Efficient media transmission	More battery drain
[75,76,77]	Smart healthcare, Biometric based IoT, vital sign signals	Framework and battery-aware	Efficient lifecycle management	Less energy saving
[78,79,80,81,82,83]	EEG, medical IoT, Secure Telemedicine and CPS	Optimal resource allocation	QoS monitoring and management	More energy and battery drain
[84,85,86,87,88,89,90]	EEG, healthcare, Ubiquitous secure and digital based	TPC based and framework	Novel ECG monitoring algorithm and framework	More battery drain
[91,92,93,94,95,96,97,98]	Smart and Green systems, EEG, Security	Routing protocols and framework	Routing and battery-based	More energy dissipation
[99,100,101,102,103,104,105,106,107,108,109,110]	Smart healthcare, Cryptography and privacy	TPC-aware	Novel Framework and method	High battery drain
[111,112,113,114]	BCI, EEG datasets	EDF tool for data	Performance metrics	RF classifier

**Table 2:** Experimental analysis of EEG data by using Random Forest classifier.

Subject	Accuracy	TP Rate	Precision	AUC
S1	86.01%	0.86	0.902	0.956
S2	67.3%	0.670	0.654	0.836
S3	90.01%	0.90	0.951	0.956
S4	96.11%	0.961	0.961	0.987
S5	64.41%	0.644	0.65	0.713
S6	62.34%	0.623	0.624	0.81
S7	60.12%	0.601	0.62	0.713
S8	89.37%	0.894	0.892	0.951
S9	84.11%	0.841	0.80	0.930
S10	99.43%	0.994	0.965	1
S11	95.73%	0.96	0.965	0.988
S12	88.41%	0.884	0.815	0.875
S13	60.17%	0.602	0.601	0.80
S14	61.38%	0.614	0.602	0.780
S15	59.44%	0.594	0.673	0.804
S16	95.31%	0.953	0.954	0.985
S17	89.43%	0.894	0.910	0.976
S18	90.37%	0.904	0.900	0.968
S19	61.42%	0.614	0.601	0.801
S20	98.31%	0.983	0.983	0.976

## Data Availability

Datsets from one of the online database, available online: https://physionet.org/ (accessed on 13 January 2022).
